# Promoting Effects of *Piriformospora indica* on the Growth and Development of Asparagus (*Asparagus officinalis* L.) Seedlings

**DOI:** 10.3390/plants14081232

**Published:** 2025-04-17

**Authors:** Jing Zhao, Ying Wang, Huixia Song, Chao Luo, Chunzhen Cheng, Liping Mao

**Affiliations:** College of Horticulture, Shanxi Agricultural University, Jinzhong 030801, China; zhaojing1@sxau.edu.cn (J.Z.); wy11332024@163.com (Y.W.); s2274341943@163.com (H.S.); 17760901351@163.com (C.L.)

**Keywords:** asparagus seedlings, *Piriformospora indica*, fungal colonization, plant growth-promoting effects, root development

## Abstract

As an endophytic fungus, *Piriformospora indica* has attracted great attention for its plant growth- and stress resistance-promoting effects on various host plants. However, up until now, there have been no reports on its application in asparagus. In this study, we report the colonization ability of *P. indica* in the roots of three asparagus varieties, ‘Guanjun’ (GJ), ‘Fengdao No. 2’ (FD), and ‘Jin Lusun No. 1’ (JL), with colonization ratios of 80.0%, 76.6%, and 73.3%, respectively. The influences of this fungal colonization on the growth of GJ, FD, and JL seedlings were further studied by determining the growth- and phytohormone-related parameters. The results showed that, at 2 months post inoculation (mpi), the *P. indica*-colonized seedlings exhibited improved total root length, peroxidase (POD) activity, and jasmonic acid (JA) accumulation in their roots and photosynthetic pigment accumulation in the leaves of all three varieties. At 8 mpi, most of the detected growth-related parameters, such as plant height, stem number and width, dry weight, photosynthetic pigment accumulation, and POD activity, were improved by the fungal colonization. However, the contents of 1-aminocyclopropane-1-carboxylic acid (ACC) in the *P. indica*-colonized roots were lower than that in the non-colonized ones. Moreover, the fungus’s promoting effects on GJ were found to be the best of the three varieties. These results indicate that *P. indica* colonization can promote asparagus seedling growth and development by enhancing root development and by regulating phytohormone balance, with some variety-specific and temporal differences.

## 1. Introduction

*Piriformospora indica* (also called *Serendipita indica*), an endophytic fungus isolated from the Indian Thar desert [1,2], is an arbuscular mycorrhizal fungus (AMF)-like fungus. Compared with AMF, *P. indica* has two main advantages: (1) it can form symbiotic relationships with more plants (with a broader host range that encompasses plants from over 30 families [3]), and (2) it can be cultured on artificial media [4,5]. Evidence has revealed that its fungal colonization can promote the growth and development of its host plants [6], improve crop yield and quality [7,8], and enhance the host plants’ resistance to various abiotic and biotic stresses [9,10,11], making it a beneficial fungus with great application prospects.

Previous studies showed that the plant growth-promoting effects of *P. indica* can be achieved through several means, such as improving root nutrient absorption and utilization abilities, mediating secondary metabolites and phytohormone accumulations [12], enhancing photosynthetic efficiency [13,14], regulating the physical–chemical states of both plants and the soil [6,12], and so on. In some host plants, the fungal promoting effects were achieved by influencing host plants from multiple aspects. For example, in longan, *P. indica* colonization enhances root development and plant resistance [13]. It improves rooting ability by inducing indoleacetic acid (IAA) biosynthesis and enhancing peroxidase (POD) activity. It also accelerates root growth by reducing H_2_O_2_ and jasmonic acid (JA) accumulation. Additionally, it boosts plant resistance to environmental stresses by increasing flavonoids levels in the stems and leaves [13].

*P. indica* colonization can improve the growth parameters of barley [15], tobacco [16], and maize [17] and can decrease the sterility of rice spikelet [18]. The promoting effects of *P. indica* colonization on root POD activity, an important rooting ability indicator, have been identified in a lot of plant species, including strawberry [19], banana [20], *Brassica campestris* [21], and so on. This fungal colonization also greatly influences the phytohormone metabolism and signaling in host plant roots. Increases in IAA accumulation in the roots after *P. indica* colonization have been found in upland rice [22] and blueberry [3]. Moreover, *P. indica* colonization has been shown to reduce ethylene or its precursor, 1-aminocyclopropane-1-carboxylic acid (ACC), in roots, which is beneficial for plant root development [3,23].

Asparagus, a perennial plant belonging to the *Asparagus* genus of the Asparagaceae family, is native to the eastern coast of the Mediterranean Sea and Asia Minor [24]. Given its high levels of polysaccharides, flavonoids, phenolic compounds, and steroid saponins [25,26,27,28], asparagus can be effectively incorporated into a variety of healthcare and pharmaceutical products. As the largest asparagus producer globally, China has a cultivated area of approximately 100,000 hectares. Moreover, the asparagus produced in China is of excellent quality due to the quite suitable climate environment [29]. However, the slow growth during its early seedling stage makes the asparagus seedling period longer than that of most other vegetables. Therefore, enhancing seedling growth and shortening the seedling period can greatly benefit the asparagus industry. It is noted that AMF inoculation can increase the biomass, photosynthetic efficiency, and nutrient (especially rutin and saponin) accumulation of asparagus plants [30,31]. As *P. indica* is an AMF-like fungus, we speculate that it may have similar promoting effects on asparagus. To verify this speculation, we first confirmed the colonization ability of *P. indica* in the roots of three asparagus varieties: ‘Guanjun’ (GJ, the most widely planted variety), ‘Fengdao No. 2’ (FD, a high-yielding variety), and ‘Jin Lusun No. 1’ (JL, a high-yield, high-quality variety). Additionally, we investigated the influences of *P. indica* colonization on the growth and development of seedlings of the three varieties. The parameters measured included plant height and biomass; stem number; chlorophyll and carotenoid contents in the leaves; IAA, JA, and ACC contents in the roots; POD activity in the roots; root vitality; and root architecture-related parameters. These measurements were conducted at 2 and 8 months post inoculation (mpi). Our study can provide a basis for future applications of *P. indica* in asparagus cultivation.

## 2. Results

### 2.1. Effects of P. indica on the Growth and Development of Asparagus Seedlings

At 30 days post *P. indica* inoculation, asparagus root samples were collected and subjected to Trypan Blue staining (Supplemental Figure S1). The results revealed that the fungal colonization ratios in the roots of GJ, FD, and JL were 80.0%, 76.6%, and 73.3%, respectively. These results indicated that *P. indica* could colonize asparagus roots, and its colonization ratio varies slightly among different varieties.

**Figure 1:**
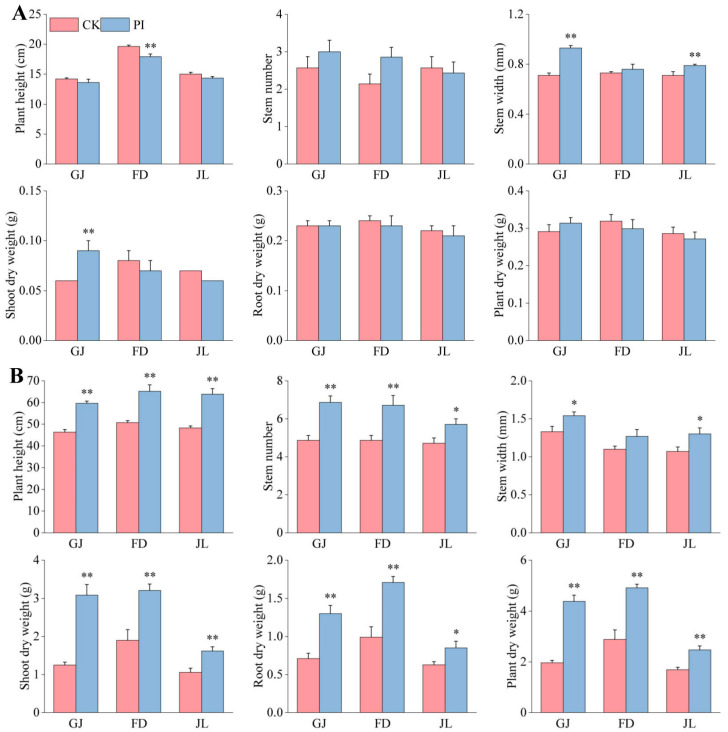
Effects of *P. indica* colonization on the growth and development of ‘Guanjun’ (GJ), ‘Fengdao No. 2’ (FD), and ‘Jin Lusun No. 1’ (JL) seedlings. (**A**) At 2 months post inoculation; (**B**) at 8 months post inoculation. CK: non-colonized controls, PI: *P. indica*-colonized plants. ‘*’ and ‘**’ indicate significant differences (*p* < 0.05) and very significant differences (*p* < 0.01) between CK and PI plants, respectively. Bars above columns represent the standard errors (SEs).

At 2 mpi, we found that the influences of *P. indica* colonization on different asparagus varieties varied (Figure 1). The promoting effects of *P. indica* on GJ were found to be the best. The fungal colonization very significantly improved its stem width and shoot dry weight (*p* < 0.01) (Figure 1A). Although the fungal colonization increased the stem number of FD, its plant height was significantly decreased. Moreover, the fungal colonization showed very significant increases in the stem width of JL (*p* < 0.01) (Figure 1A).

**Figure 2:**
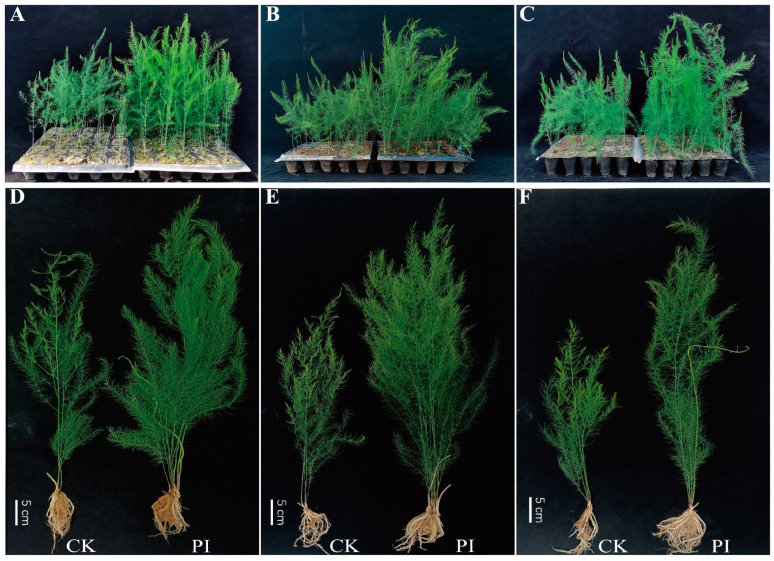
Effects of *P. indica* colonization on the phenotypes of ‘Guanjun’ (**A**,**D**), ‘Fengdao No. 2’ (**B**,**E**), and ‘Jin Lusun No. 1’ (**C**,**F**) asparagus seedlings. (**A**–**C**) At 6 months post inoculation; (**D**–**F**) at 8 months post inoculation. In (**A**–**F**), non-colonized control seedlings are on the left, and *P. indica* colonized seedlings are on the right. CK: non-colonized control, PI: *P. indica*-colonized.

At 6 mpi (Figure 2A–C) and 8 mpi (Figure 2D–F), *P. indica* colonization obviously improved the plant height of all three varieties. At 8 mpi, all the growth-related parameters were found to be improved by *P. indica* (Figure 1B and Figure 2). The plant height, stem number, shoot and root dry weight, and plant dry weight of all three varieties were significantly (*p* < 0.05) or very significantly (*p* < 0.01) improved by the fungal colonization. Notably, the plant dry weight of GJ, FD, and JL increased very significantly, by 123.46%, 72.40%, and 45.64% (*p* < 0.01), respectively.

### 2.2. Influences of P. indica Colonization on Root Architecture Parameters of Asparagus Seedlings

**Figure 3:**
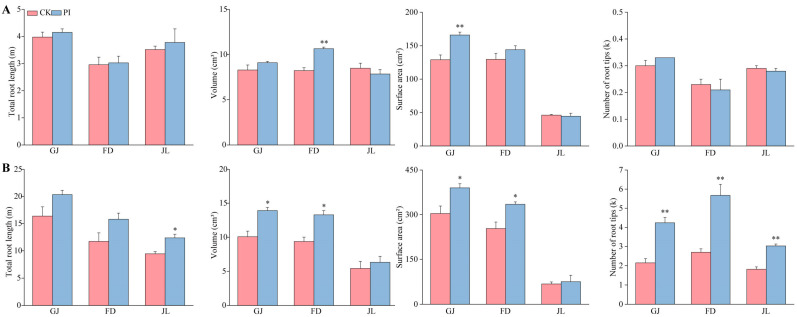
Influences of *P. indica* colonization on root architecture parameters of ‘Guanjun’ (GJ), ‘Fengdao No. 2’ (FD), and ‘Jin Lusun No. 1’ (JL) seedlings. (**A**) At 2 months post inoculation; (**B**) at 8 months post inoculation. CK: non-colonized control, PI: *P. indica*-colonized; ‘*’ and ‘**’ indicate significant differences (*p* < 0.05) and very significant differences (*p* < 0.01) between PI and CK of each asparagus variety, respectively. Bars above columns represent the standard errors (SEs).

The influences of *P. indica* colonization on the asparagus root architecture were further investigated (Figure 3). At 2 mpi, the fungal colonization very significantly improved the root surface area of GJ (accounting for 1.21-fold of CK), and the root volume of FD (approximately 1.29-fold of CK) (*p* < 0.01) (Figure 3A).

At 8 mpi, the root growth-promoting effects of the fungal colonization were more significant (Figure 3B). In all three asparagus varieties, the PI seedlings exhibited higher values of total root length, volume, surface area, and root tip number compared to their corresponding control seedlings. Notably, the root tip numbers of all the PI seedlings were very significantly improved (*p* < 0.01), accounting for 1.97-, 2.10-, and 1.66-fold of their corresponding CK for GJ, FD, and JL, respectively. Moreover, the volume and surface area of the *P. indica*-colonized GJ and FD roots were significantly higher than their corresponding CK (*p* < 0.05). For JL, the total root length of the PI seedlings was significantly improved (*p* < 0.05), accounting for approximately 1.31-fold of CK.

### 2.3. Effect of P. indica on Root Vitality and POD Activity of Asparagus Seedlings

**Figure 4:**
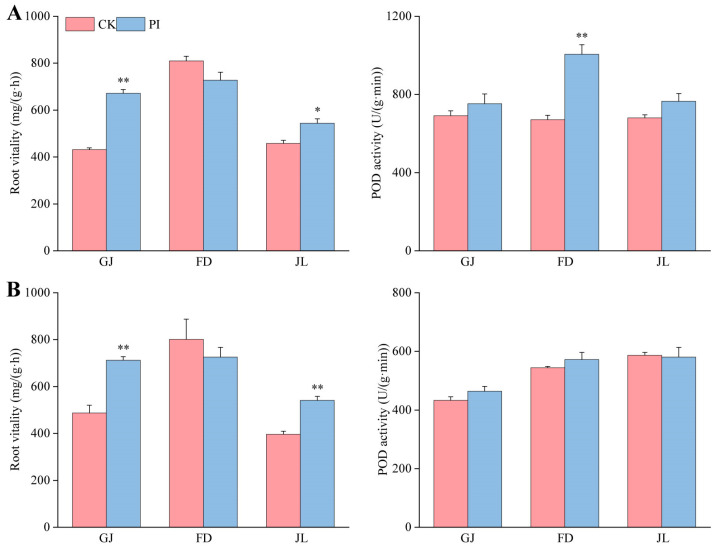
Influences of *P. indica* colonization on the root vitality and root POD activity of ‘Guanjun’ (GJ), ‘Fengdao No. 2’ (FD), and ‘Jin Lusun No. 1’ (JL) seedlings. (**A**) At 2 months post inoculation; (**B**) at 8 months post inoculation; CK: non-colonized control, PI: *P. indica*-colonized. ‘*’ and ‘**’ indicate significant differences (*p* < 0.05) and very significant differences (*p* < 0.01) between PI and CK plants, respectively. Bars above columns represent the standard errors (SEs).

We further determined the root vitality and POD activity (an important rooting ability indicator) in asparagus roots. *P. indica* colonization significantly improved the root vitality of GJ at both 2 mpi (Figure 4A) and 8 mpi (Figure 4B) (*p* < 0.05), accounting for 1.56- and 1.46-fold of their corresponding CK, respectively. The fungal colonization significantly improved the root vitality of JL at 2 mpi (Figure 4A) (*p* < 0.05) and very significantly improved its root vitality at 8 mpi (Figure 4B) (*p* < 0.01). However, *P. indica* colonization showed no significant influence on the root vitality of FD, which might be related to its high basal root vitality.

At 2 mpi, *P. indica* colonization increased POD activities in the roots of all three varieties. Notably, very significant POD activity increases were identified in the *P. indica*-colonized FD roots (Figure 4A), accounting for 1.50-fold of CK. At 8 mpi, no significant POD activity difference was identified between CK and PI of all three asparagus seedlings (Figure 4B).

### 2.4. Effects of P. indica on Photosynthetic Pigment Accumulation in the Leaves of Asparagus Seedlings

**Figure 5:**
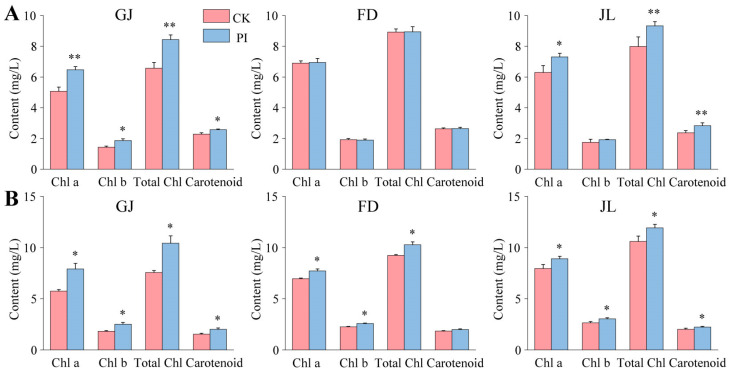
Effects of *P. indica* on photosynthetic pigment accumulation in the leaves of ‘Guanjun (GJ)’, ‘Fengdao No. 2’ (FD), and ‘Jin Lusun No. 1’ (JL). (**A**) At 2 months post inoculation; (**B**) at 8 months post inoculation. Chl: chlorophyll; CK: non-colonized control; PI: *P. indica*-colonized. ‘*’ and ‘**’ indicate significant differences (*p* < 0.05) and very significant differences (*p* < 0.01) between CK and PI plants, respectively. Bars above columns represent the standard errors (SEs).

The photosynthetic pigment contents in the leaves of the CK and PI asparagus seedlings were also determined. At 2 mpi, the fungal colonization showed very significant increases in the chlorophyll a and total chlorophyll contents in the leaves of GJ (*p* < 0.01), both accounting for 1.28-fold of their corresponding CK. The fungal colonization significantly increased the chlorophyll b and carotenoid contents in the GJ leaves (*p* < 0.05), accounting for 1.31- and 1.13-fold of their corresponding CK (Figure 5A), respectively. The fungal colonization significantly increased the chlorophyll a content (*p* < 0.05) and very significantly increased the total chlorophyll and carotenoid contents (*p* < 0.01) in the JL leaves (Figure 5A). However, no significant difference was identified between the CK and PI of FD (Figure 5A).

At 8 mpi, in all three varieties, the chlorophyll a, chlorophyll b, total chlorophyll, and carotenoid contents in the leaves of the PI seedlings were significantly higher than those of their corresponding CK (*p* < 0.05) in all three varieties, except for the carotenoid content in FD (Figure 5B). The chlorophyll a, chlorophyll b, total chlorophyll, and carotenoid contents in the leaves of *P. indica*-colonized GJ were all significantly increased (*p* < 0.05) by approximately 30% compared to those in CK (Figure 5B). For FD, the chlorophyll a, chlorophyll b, and total chlorophyll contents were significantly higher in PI (*p* < 0.05), accounting for 1.11-, 1.14-, and 1.11- fold of CK, respectively (Figure 5B). The chlorophyll a, chlorophyll b, total chlorophyll, and carotenoid contents in the PI leaves of JL were all significantly higher (*p* < 0.05) (Figure 5B), accounting for 1.12-, 1.15-, 1.13-, and 1.11-fold of CK, respectively.

### 2.5. Effects of P. indica on IAA, JA, and ACC Contents in the Roots of Asparagus Seedlings

**Figure 6:**
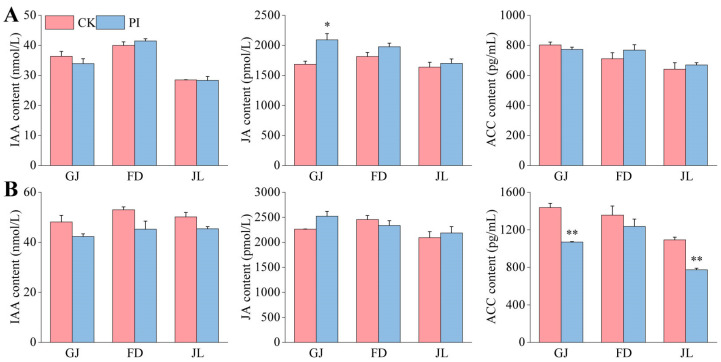
Effects of *P. indica* on IAA, ACC, and JA contents in the roots of ‘Guanjun’ (GJ), ‘Fengdao No. 2’ (FD), and ‘Jin Lusun No. 1’ (JL) seedlings. (**A**) At 2 months post inoculation; (**B**) at 8 months post inoculation, CK: non-colonized control, PI: *P. indica*-colonized. ‘*’ and ‘**’ indicate significant differences (*p* < 0.05) and very significant differences (*p* < 0.01) between CK and PI plants, respectively. Bars above columns represent the standard errors (SEs).

To investigate the effects of *P. indica* on phytohormone accumulation in asparagus roots, we determined the IAA, JA, and ACC contents. The fungal colonization had no significant effect on IAA content in the roots of the three varieties both at 2 mpi and 8 mpi (Figure 6A,B).

At 2 mpi, the JA contents in the *P. indica*-colonized roots of all three asparagus varieties were found to be higher than those in their corresponding CK (Figure 6A). The JA content in the *P. indica*-colonized GJ roots was significantly higher (*p* < 0.05) than that in its controls, accounting for approximately 1.24-fold of CK (Figure 6A). At 8 mpi, the JA content in the roots of the CK and PI seedlings did not differ significantly (Figure 6B).

At 2 mpi, no significant root ACC content difference was identified between CK and PI of all three asparagus varieties (Figure 6A). At 8 mpi, however, the ACC contents in the *P. indica*-colonized GJ, FD, and JL roots were all lower than those in their corresponding CK (Figure 6B). It should be noted that the ACC content in the roots of *P. indica*-colonized GJ and JL very significantly decreased by 25.7% and 29.1% (*p* < 0.01), respectively.

## 3. Discussion

### 3.1. P. indica Promotes the Growth of Asparagus Seedlings

As an endophytic fungus, the host range of *P. indica* is extremely wide [32]. In the last decade, more and more horticultural plants and crops have been confirmed to be able to form symbiosis with *P. indica*, including longan [13], gerbera [14], strawberry [19], date palm [33], passion fruit [34], sweet potato [35], and so on. In this study, we found that *P. indica* can successfully colonize asparagus roots, achieving a colonization rate > 70% after three rounds of *P. indica* suspension irrigation in all three varieties. These findings indicate that *P. indica* can easily colonize asparagus roots and establish symbiotic relationship with asparagus. Although the growth-promoting effects of the fungus were less evident at 2 mpi, our findings reveal that the fungal colonization significantly promotes the seedling growth of the three asparagus varieties at 8 mpi. This indicates that *P. indica* has great potential to be applied in asparagus seedling cultivation.

*P. indica* colonization substantially enhances root vitality, root number, and root surface area in host plants [13,14]. These changes can enhance nutrient and water absorption efficiency, thereby stimulating the growth of host plant above-ground parts [11,20]. In this study, we found that *P. indica* colonization significantly improved most of the root architecture-related parameters in all three asparagus varieties at both 2 mpi and 8 mpi. Consistently, at 2 mpi, root POD activity (a marker for rooting ability [3]) was enhanced in all three varieties, and root vitality was improved in GJ and JL. These findings suggest that, although the growth-promoting effects of the fungus were less evident at 2 mpi than at 8 mpi, the fungal colonization enhanced asparagus root development in the early stage.

Evidence revealed that *P. indica* colonization can increase chlorophyll accumulation in the leaves of host plants, such as *Anthurium* [36], soybeans [37], and sunflower [38]. Our present study found that the fungal colonization greatly induced chlorophyll a and total chlorophyll accumulation in the leaves of GJ and JL at 2 mpi, suggesting that *P. indica* colonization enhanced their photosynthetic abilities. With the increase in root vitality and photosynthetic ability, the development and growth of plants was accelerated and finally resulted in significantly improved plant biomass of asparagus seedlings. Moreover, the fungus’s promoting effects on GJ were found to be the best of the three varieties.

### 3.2. P. indica Colonization Influences Phytohormone Accumulation in Asparagus Roots

*P. indica* can influence plant growth, flowering time, and differentiation and enhance partial and systemic immune responses by modulating phytohormone biosynthesis and signal transduction [12]. JA has been recognized as a main regulatory factor regulating adventitious root formation by inhibiting the elongation of primary roots and promoting the generation of adventitious roots [39]. Our study showed that the JA contents in *P. indica*-colonized asparagus roots were higher than those of the non-colonized controls at 2 mpi, with a significant increase in GJ roots. Moreover, the JA contents in GJ and JL roots were also higher than those of their corresponding CK at 8 mpi, accompanied by increased root tip number, root volume, and root biomass.

IAA has the functions of regulating the growth rate of stems, inhibiting lateral bud differentiation, promoting rooting, and so on. *P. indica*-colonized upland rice [22], *Cerasus humilis* [23], and blueberry [3] plants accumulated more IAA in their roots compared to their non-colonized controls. However, the influences of *P. indica* colonization on IAA accumulation are not the same in different host plants. For example, although *P. indica* colonization promoted the growth of Arabidopsis plants, the IAA concentration in their roots reduced [40]. The biomass of *P. indica*-colonized *Pinus elliottii* significantly increased, but no significant IAA content change was identified [41]. Our study showed that, although the IAA contents showed no significant changes at both 2 mpi and 8 mpi, the plant dry weight and stem numbers of the PI seedlings significantly increased at 8 mpi. These indicate that the growth-promoting effects of *P. indica* in asparagus were not achieved by improving IAA biosynthesis.

Ethylene, a gaseous phytohormone synthesized from the precursor ACC, is involved in multiple growth and developmental processes of plants [42]. ACC functions as a growth regulator and has a negative impact on root growth [43,44]. *P. indica* colonization can significantly reduce the ACC content and increase the biomass of *C. humilis* cuttings [23]. The ethylene biosynthesis and signal transduction in barley roots were greatly influenced by *P. indica* colonization [45]. In barley, although most ethylene biosynthesis genes are upregulated during the *P. indica* colonization process, the ethylene signal components are typically suppressed [46]. In this study, the ACC contents in the *P. indica*-colonized roots of all three asparagus varieties were lower than those of their corresponding controls at 8 mpi, suggesting that *P. indica* can decrease the ACC content in the roots of asparagus to promote rooting.

**Figure 7:**
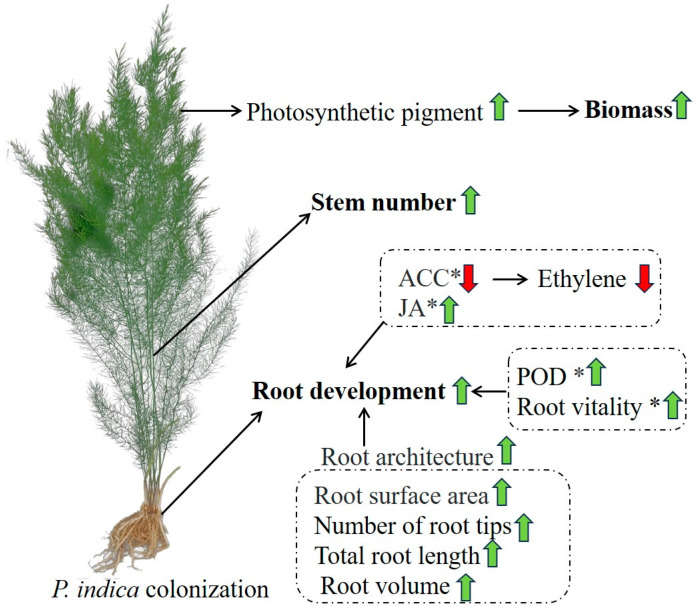
Summary diagram for the growth-promoting effects of *P. indica* on asparagus. POD: peroxidase; ACC: 1-aminocyclopropane-1-carboxylic acid; JA: jasmonic acid. Black arrows represent promoting effects. Green and red arrows represent upregulation and downregulation of parameters, respectively. ‘*’ indicates that this effect varied among different asparagus varieties.

Collectively, our study revealed the physiological and biochemical mechanisms underlying the promoting effects of *P. indica* on asparagus seedlings (Figure 7): (1) *P. indica* colonization improved the root vitality, POD enzyme activity, root tip number, total root length, root volume, and surface area of asparagus plants, thereby enhancing its root architecture and promoting root development; (2) the fungal colonization decreased the contents of ACC but increased the JA content in asparagus roots, which was helpful for the root development of asparagus seedlings; and (3) the fungal colonization improved the photosynthetic pigment accumulation in the leaves, which promoted their photosynthetic ability and biomass accumulation and increased the stem number of asparagus seedlings.

## 4. Materials and Methods

### 4.1. Plant Materials and Fungal Inoculation

The seeds of ‘Guanjun’ (GJ) asparagus were purchased from Weifang Academy of Agricultural Sciences (Weifang, China), the seeds of ‘Fengdao No. 2’ (FD) were purchased from Hangzhou Zhefeng Technology Co., Ltd. (Hangzhou, China), and the seeds of ‘Jin Lusun No. 1’ (JL) were provided by our research group. The *P. indica* strain (DSM11827) was provided by professor KaiWun-Yeh of Taiwan University (Taiwan, China) and kept in our lab.

Full seeds of the three asparagus varieties were selected for disinfection by soaking in 55~58 °C water for 30 min and soaked at 25 °C for 72 h. Then, the seeds were sown in substrates in 50-hole trays for seedling cultivation in a simple temperature-controlled greenhouse with day temperatures of 25~30 °C and night temperatures of 15~20 °C from August to October. The substrate used for seedling cultivation was purchased from Shen County Luyuan Seedling Matrix Co., Ltd. (Liaocheng, China), with an organic matter content of ≥35% and a neutral pH. For each variety, three trays of seedlings were cultivated. In this stage, the seedlings were irrigated every day with 1/4 Hoagland nutrient solution (Phygene Life Sciences Co., Ltd., Fuzhou, China) and water alternately.

According to the method described by Cheng et al. [47], the fungal solution used for inoculation was prepared and adjusted to 2 × 10^7^ spores/mL using potato glucose broth medium (PDB, Hope Bio-Technology Co., Ltd., Qingdao, China). *P. indica* inoculation was performed at 20 days after sowing. Seedlings of one tray were irrigated with 1 L of fungal solution (PI) every five days, for a total of three times. Seedlings of the control group (CK) were irrigated with an equal amount of water. The *P. indica* inoculation experiment was performed in September 2023. As asparagus seedlings should experience dormancy in winter, the temperature of the simple temperature-controlled greenhouse was set at natural temperatures for about four months post *P. indica* inoculation until the end of February 2024. In March 2024, the temperature of the greenhouse was set back to day temperatures of 25~30 °C and night temperatures of 15~20 °C. The seedlings were cultivated in the original trays for about one month and then transplanted into pots (diameter 12.5 cm × 9.2 cm) containing the same matrix for another two months. The seedlings were irrigated every two or three days with 1/2 Hoagland nutrient solution and water alternately.

### 4.2. P. indica Colonization Observation

At 30 days post inoculation, all *P. indica* solution-irrigated asparagus roots were subjected to Trypan Blue staining [19]. The asparagus roots were collected, washed with sterile water, and cut into about 1 cm segments. Then, the root segments were immersed in a 10% KOH solution for 24 h, rinsed with pure water three times, immersed in a 1% HCl solution for 10 min, rinsed with pure water three times, stained with 0.05% Trypan Blue solution for 20 min, mounted on slides, and observed and photographed under an optical microscope (Eclipse Ni-U, NIKON Corporation, Tokyo, Japan). For each seedling, three root segments were observed. The fungal colonization ratio for each variety was calculated according to the following formula: colonization ratio (%) = number of root segments with *P. indica* colonization/total number of root segments × 100% [14]. For the CK asparagus seedlings, 30 root segments for each variety were used for Trypan Blue staining and used as controls.

### 4.3. Determination of Plant Growth-Related Parameters

Plant samples were randomly selected from the PI and CK groups of the three asparagus varieties and washed with distilled water to remove any attached matrix. The plant height, stem number, and stem width of the plants were measured with at least 7 biological replications. The dry weight of the above seedlings was measured after drying at 105 °C for 10 min and then at 80 °C to constant weight.

### 4.4. Determination of Root Architecture-Related Parameters

Five seedlings were randomly selected from the PI and CK groups of the three asparagus varieties for the determination of the root architecture-related parameters. The roots were washed with distilled water to remove any attached substrates and then scanned using Scan Wizard EZ (GXY-A, Zhejiang Top Cloud Agri Technology Co., Ltd., Hangzhou, China). Then, the WinRHIZ V2.3.2 root analysis system was used to calculate the total root length, total root surface area, total root volume, and total root tips of the seedlings.

### 4.5. Determinations of Leaf Photosynthetic Pigment, Root Vitality, and Root POD Activity

To determine the leaf photosynthetic pigment contents, root vitality and root POD activity, six seedlings were randomly selected from the PI and CK groups of the three asparagus varieties. The contents of the leaf photosynthetic pigment, including chlorophyll a, chlorophyll b, total chlorophyll, and carotenoid, were determined by the ethanol-acetone extraction method [13].

The root vitality was determined by using the TTC reduction method [23]. The root POD activity was determined by using the guaiacol colorimetry method [48].

### 4.6. Measurements of Phytohormone Contents in the Roots

Six seedlings were randomly selected from the PI and CK groups of the three asparagus varieties for the determinations of the phytohormone contents in the roots. The content of IAA, JA, and ACC in the asparagus roots were determined using the enzyme-linked immunosorbent assay (ELISA) method according to the manuals of commercial kits produced by Shanghai Tongwei Co., Ltd. (Shanghai, China)

### 4.7. Statistical Analysis

The data obtained in this study were analyzed by Student’s *t*-test at 5% and 1% levels using SPSS 25.0 statistical analysis software. All results are displayed as the ‘mean (standard error, SE)’ of at least three replications. Origin Pro 2024 software was used for figure drawing.

## 5. Conclusions

In this study, for the first time, we demonstrated the colonization ability of *P. indica* in asparagus roots. The fungal colonization significantly promotes the seedling growth of the three asparagus varieties at 8 mpi. At 2 mpi, although the effects of *P. indica* colonization on the growth of asparagus aboveground parts were not obvious, the fungal colonization enhanced root development. Moreover, the fungus’s growth-promoting effects varied among different varieties. This study can provide a basis for the future application of *P. indica* in asparagus cultivation.

## Data Availability

Data are contained within the article and Supplementary Materials.

## Supplementary Materials

The following supporting information can be downloaded at https://www.mdpi.com/article/10.3390/plants14081232/s1, Figure S1: Colonization detection results of *P. indica* in asparagus roots. (A–C) *P. indica*-colonized ‘Guanjun’, ‘Fengdao No.2’, and ‘Jin Lunsun No.1’ roots. (D) Non-colonized control roots. The black arrows point to the *P. indica* chlamydospores, and the red arrows point to *P. indica* hyphae.
