# Supplementary figures and images for: Promoting Effects of Piriformospora indica on the Growth and Development of Asparagus (Asparagus officinalis L.) Seedlings
Source: Plants (Basel). 2025 Apr 17;14(8):1232. doi: 10.3390/plants14081232 (PMC12030300; doi:10.3390/plants14081232)

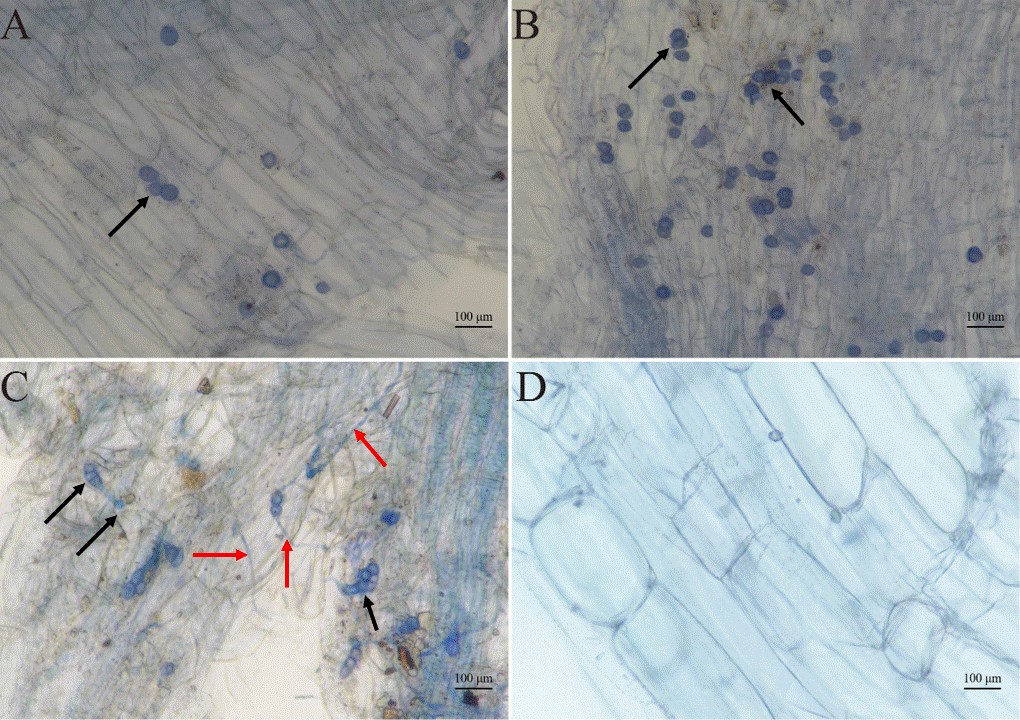

Supplement: Supplementary file 1 [file plants-14-01232-s001.zip › plants-3414937-supplementary.jpg]
